# EGFR modulates complement activation in head and neck squamous cell carcinoma

**DOI:** 10.1186/s12885-020-6615-z

**Published:** 2020-02-13

**Authors:** Anas H. A. Abu-Humaidan, Lars Ekblad, Johan Wennerberg, Ole E. Sørensen

**Affiliations:** 1 Division of Infection Medicine, Department of Clinical Sciences, Lund University, Lund, Sweden; 2 Division of Microbiology, Faculty of Medicine, The University of Jordan, Amman, Jordan; 3 Division of Oncology, Department of Clinical Sciences, Lund University, Lund, Sweden; 4 Division of Otorhinolaryngology/H&N Surgery, Department of Clinical Sciences, Lund University, Lund, Sweden

**Keywords:** EGFR, HNSCC, Complement activation, Complement regulation, Tyrosine kinase inhibitor, Iressa, Cancer microenvironment

## Abstract

**Background:**

The epidermal growth factor receptor (EGFR) is pivotal for growth of epithelial cells and is overexpressed in several epithelial cancers like head and neck squamous cell carcinoma (HNSCC). EGFR signalling is also involved in diverse innate immune functions in epithelia. We previously found a role for EGFR in modulating the complement system in skin, this prompted an investigation into EGFR role in complement modulation in HNSCC.

**Methods:**

We used patient derived HNSCC cell lines with varying sensitivities to EGFR inhibitors, and generated EGFR inhibition resistant cell lines to study the role of EGFR in modulating complement in HNSCC.

**Results:**

We found that HNSCC cell lines activate the complement system when incubated with human serum. This complement activation was increased in cell lines sensitive to EGFR inhibition following the use of the tyrosine kinase inhibitor Iressa. Sensitive cell line made resistant to EGFR-inhibitors displayed complement activation and a decrease in complement regulatory proteins even in the absence of EGFR-inhibitors. Complement activation did not cause lysis of HNSCC cells, and rather led to increased extracellular signal-regulated kinase (ERK) phosphorylation in one cell line.

**Conclusion:**

These data indicate that EGFR has a complement modulatory role in HNSCC, and that a prolonged EGFR-inhibition treatment in sensitive cancer cells increases complement activation. This has implications in understanding the response to EGFR inhibitors, in which resistance and inflammatory skin lesions are two major causes for treatment cessation.

## Background

The complement system is a conserved cornerstone of innate immunity [1, 2]. More than 30 proteins comprising the complement system play a role in various immune and homeostatic functions, from killing of pathogens and clearance of apoptotic cells [3–6], to recently discovered roles in angiogenesis and tissue regeneration [7–9]. Hence, activation of the complement system is tightly regulated both at the level of initiation and amplification [10–12]. An important part of this regulation occurs locally in tissue [13], indeed, almost all nucleated cells express at least one complement regulator on their surface [14]. This local regulation is highlighted by tissue specific manifestations of systemic complement deficiencies [12, 15, 16].

The role of the complement system in cancer development is unclear [17], but data show complement activation in the microenvironment of several epithelial cancers [18–20], and elevated levels of activation fragments in serum of cancer patients [18, 20, 21]. Complement activation has been traditionally seen as an immune surveillance mechanism against cancer development [22]. Consequently, antibody therapies aim to promote complement dependent cytotoxicity [23], but malignant cells tend to upregulate the expression of complement regulatory proteins [24], highlighting a selective pressure exerted on these cells to minimize the harmful effects of complement.

On the other hand, several studies demonstrate a cancer promoting effect of complement activation fragments, either through recruitment of immunomodulatory cells like myeloid-derived suppressor cells [25], or by directly interacting with receptors (e.g. C3aR, C5aR) that activate growth signalling pathways like ERK1/2, or EGFR transactivation [26, 27]. In many carcinomas, local complement expression and activation promote cancer growth [28]. The finding that C5aR inhibition retarded cancer growth in mice to a similar extent exhibited by anticancer drugs [25] warrants more detailed studies of complement regulatory mechanisms in the cancer microenvironment.

EGFR inhibition therapy is frequently used in treatment of HNSCC [29], and inhibition of EGFR signalling in epithelial tissues induces a local inflammatory environment [30]. An inflammatory environment is integral for the neoplastic process in general [31], and even more so in HNSCC [32–34]. Interestingly, although inflammation is beneficial for growth of HNSCC, EGFR inhibition-induced inflammatory skin lesions during treatment is the best predictor for treatment response. We previously found that EGFR regulated complement expression in primary keratinocytes, and consequently, we now sought to examine if EGFR has a role in regulation of complement activation in HNSCC.

In this study we found that EGFR inhibition treatment induced complement activation on the cell surface of cancer cells. Complement activation did not cause cell lysis but rather increased ERK phosphorylation for one of the cell lines tested.

## Materials and methods

### Reagents

Iressa was purchased from Sigma-Aldrich; cetuximab (Erbitux) from Merck. Affinity purified polyclonal rabbit antibodies against the C3d domain of human C3, and against the C4c domain of human C4 were from Dako. Monoclonal mouse anti C5b-9 antibody, human purified C1q, C1q-depleted serum, and factor B–depleted serum were from Quidel. Monoclonal mouse antibody against GAPDH, affinity purified rabbit antibodies against ERK and phosphorylated ERK were from R&D systems.

### Cell culture

Head and neck squamous cell carcinoma cell lines LU-HNSCC- [4, 5, 7, 8] - *referred to hereafter as HN* [4, 5, 7, 8] *in the figures* - were generated at the Divisions of Ear, nose and throat/ Head and neck Surgery and Oncology at Lund University as previously described [35, 36]. A431 (Human squamous carcinoma, ECACC no. 85090402) and A549 (Human Caucasian lung carcinoma, ECACC no. 86012804) were from Sigma. All cell lines were cultured in DMEM supplemented with 10% heat inactivated foetal bovine serum (FBS) and antibiotics (30 μg/mL Gentamicin, 15 ng/mL Amphotericin, Gibco). HN4 from the floor of the mouth, HN5 from the gingiva, HN7 from a recurrence of a squamous cell carcinoma of the bucca, and HN8 from the bucca. Primary keratinocytes were obtained from Lonza and grown in serum-free medium (KGM Gold Bullet Kit) from Lonza. For 2–4 d after seeding, the keratinocytes received 100 ng/ml EGF. For all cell types, medium was changed to KGM Gold medium without insulin or EGF for 24 h before complement activation.

### Cetuximab resistant sublines

Cell lines HN4 and HN5 were treated with increasing cetuximab concentrations doubled every 2 weeks. Dose increase was performed by splitting the cells at the lower concentration, and after 3 days the medium was changed to medium with double cetuximab concentration. The cell lines not treated with cetuximab were grown and split in the same manner as the cetuximab-treated cells. When maximum concentration for each cell line (2560 nmol/L, 0.39 mg/mL) was reached, the cells were grown for 2 months at that concentration before freezing. Growth was measured using the Sulforhodamine B colorimetric assay as described below.

Before complement experiments, these cells were passaged at least three times with several medium changes in each passage, in medium without cetuximab to avoid possible complement activation due to cetuximab.

### Iressa sensitivity assay

To measure Iressa-mediated growth inhibition of cell lines HN4, HN5, HN7 and HN8, cells were seeded at densities averaging 2.5*10^5^ cells/ well, in 12-well plates in DMEM supplemented with 10% heat inactivated FBS and antibiotics. The next day, medium was changed to KGM bullet kit without EGF or insulin, with or without 5 μmol/L or 10 μmol/L Iressa. Cell counts were done at 24 h and 48 h after Iressa treatment using 0.4% Trypan blue staining in LUNA™ Automated Cell Counter (Logo Biosystems).

### EGFR activation and inhibition

The day cells were confluent, medium was changed to KGM bullet kit without EGF or insulin (Lonza). The day after confluency, cells were treated with 10 μmol/L Iressa for 48 h in new KGM without EGF or insulin. Non-treated control cells were grown in the same medium but without treatment.

### Real-time polymerase chain reaction (R-T PCR)

cDNA was synthesized from 600 ng purified RNA using iScript cDNA synthesis kit (Bio-Rad), according to the instructions given by the manufacturer. RNA expression of complement components was analysed with quantitative R-T PCR using iQ SYBR Green Supermix (Bio-Rad). Amplification was performed at 55 °C for 40 cycles in iCycler Thermal Cycler (Bio-Rad), and data were analyzed using iCycler iQ Optical System Software. RNA expression was normalized using GADPH as housekeeping gene.

### Complement activation

KGM medium without EGF or Insulin was added to the cells together with 10% normal human serum (NHS) or 10% heat-inactivated human serum (HIS). After 3-h incubation at 37 °C, the cells were washed with PBS and fixed with 4% formaldehyde for 1 h (15 min on ice, 45 min at room temperature). After three washes in TBS (10 mmol/L Tris, 500 mmol/L NaCl [pH 7.2]), the cells were blocked with 5% goat serum/5 mg/mL bovine serum albumin (BSA) at room temperature for 45 min in TBS. After blocking, inserts were washed once in TBS with 0,05% tween (TTBS). Primary antibodies were incubated in TTBS with 2.5% goat serum/5 mg/mL BSA overnight in at 4 °C under rotation. Slides were washed three times in TTBS and incubated with secondary Abs for 2–4 h at room temperature. After washing the inserts were mounted on slides using Prolong Gold antifade reagent mounting medium with DAPI (Invitrogen).

### Immunofluorescence microscopy

For fluorescence microscopy analysis, samples were visualized using a Nikon Eclipse TE300 (Nikon) inverted fluorescence microscope equipped with a Hamamatsu C4742–95 cooled charge-coupled device camera (Hamamatsu) and a Plan Apochromat objective (Olympus). C3, C4 and terminal complement complex (TCC) fluorescence around several fields was quantified using ImageJ, one representative experiment is shown.

### ^125^I-labeled C1q binding assay

HN4 and HN5 cells were treated with Iressa (10 μmol/L) for 48 h. Subsequently, cells were incubated with 1 μg/mL ^125^I-labeled C1q (10,000 cpm) in 3.5 mg/ml bovine serum albumin for 30 min at 37 °C. Cells were washed three times in PBS and trypsinated. The radioactivity associated with the trypsinated cells was determined in a gamma counter (PerkinElmer).

### Sulforhodamine B (SRB) assay

The colorimetric SRB assay was used to assess cell density, based on the measurement of cellular protein content [37]. Cells were washed and fixed by adding ice-cold 17% (w/v) trichloroacetic acid (TCA) to each well and incubated for 1 h at 4 °C, supernatant was discarded and plates rinsed five times with water and air-dried. Fixed cells were then stained in SRB solution (0.4% w/v SRB in 1% acetic acid) for 20 min at room temperature, rinsed five times with 1% acetic acid to remove unbound SRB and air-dried. The dye was dissolved in 150 μL 10 mmol/L Tris base and the absorbance measured at 565 nm.

### Growth assay

HN5 cells were grown to around 20% confluence. EGFR inhibition and subsequent complement activation were performed as described above. Briefly, cells were treated for 48 h with 10 μmol/L Iressa, and subsequently incubated for 3 h in 10% NHS,10% HIS or medium only. Afterwards, growth was measured using an SRB assay either directly (3 h time point) or after 21 h (24 h time point) of complement activation.

### Scratch assay

To see if complement activation promotes migration of cells, we used a modified scratch assay. The day prior to confluence of HN5 cells, medium was changed to KGM bullet kit without insulin or EGF and with 10 μmol/L Iressa. This medium was used during the remainder of the experiment. The day after confluence, a scratch was made with a 200 μL pipette tip. Immediately after the scratch, 10% NHS or HIS was added for 3 h, and pictures were taken. Medium was changed back to medium with Iressa (to minimize the effect of cell growth on the assay) and pictures were taken at 24 h from serum addition. Open areas not containing cells after the scratch were analyzed using the software TScratch [38]. Area of the scratch at 24 h was presented as a percent of the original scratch area, using the following formula: (***Open area at*** **24*h***/***Open area at*** **0*h***) ∗ **100**%, where values below 100% represent closure of the original scratch.

### SDS-PAGE and immunoblotting

SDS-PAGE and immunoblotting were performed according to the instructions from the manufacturer (Bio-Rad). After transfer of proteins from the polyacrylamide gels, the polyvinylidene difluoride (PVDF) membrane was fixed for 30 min in TBS with 0.05% glutaraldehyde (Sigma-Aldrich) and blocked with 5% BSA. For visualization of the proteins, the PVDF membranes were incubated overnight with primary antibody. The following day, the membranes were incubated for 2 h with horse radish peroxidase (HRP) conjugated secondary antibody and visualized by SuperSignal West Pico Chemiluminescent Substrate (Pierce), Quantification of signal was done using Image lab. The PVDF membrane was stripped for 20 min in 0.2 mol/L glycine (pH 2.5) and 1% SDS, washed twice with TBS with 0.05% Tween 20 (TTBS), and finally blocked before incubating overnight with a new antibody.

### ERK activation

ERK activation was monitored by quantifying the signal of Western blots, phosphorylated ERK signal was measured using chemiluminescence of and normalized to total ERK.

### Statistical analysis

Values were log-transformed, and Student’s t-test was performed on log transformed values to compare different treatments. * denotes *p* < 0.05, ** denotes *p* < 0.01.

## Results

### Head and neck squamous cell carcinoma (HNSCC) activate the complement system when incubated with human serum

**Fig. 1:**
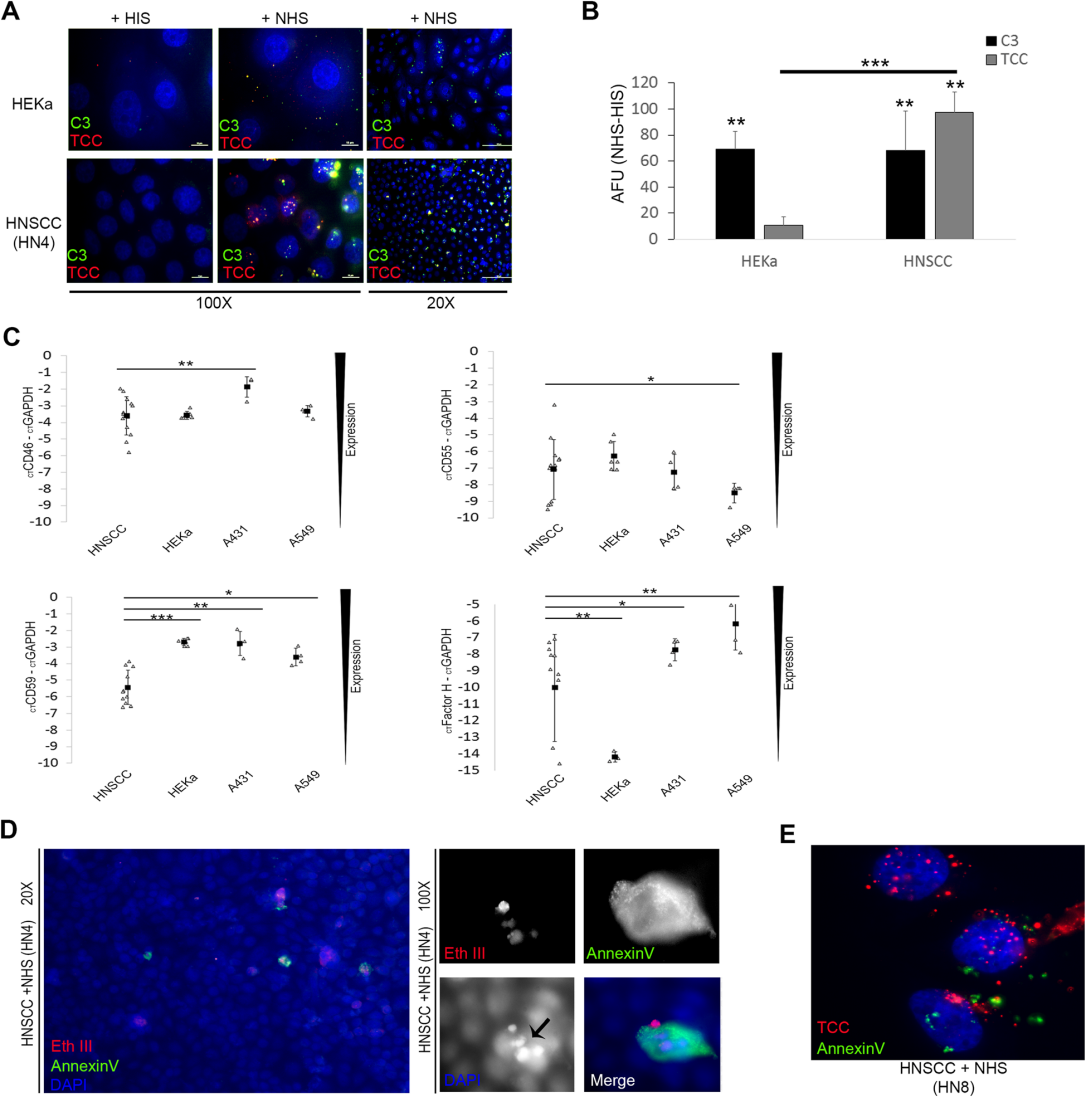
Complement deposition in viable HNSCC cell lines. (**a**) Immunofluorescence microscopy (IFM) shows deposition of complement activation fragments C3 and TCC in HNSCC cell line (HN4) when incubated with NHS but not HIS. (**b**) IFM pooled quantification of C3 and TCC deposition in 4 HNSCC cell lines was compared to HEKa cells, HNSCC showed a significant increase in TCC deposition. The quantification was done by subtracting the signal of NHS from HIS treated cells. (**c**) qPCR data of CRP CD46, CD55, CD59 and Factor H showed significantly less normalized expression of CD59 in HNSCC cell lines in comparison to other cells tested. (**d**) IFM was used to investigate apoptosis using Annexin V, and necrosis using Ethidium bromide homodimer (Eth III) in HNSCC (HN4) monolayers, very few cells stained with either. A close up of a cell that stains for apoptosis and necrosis markers shows abnormal nuclear morphology in the DAPI channel (arrow). (**e**) A double stain of TCC and Annexin V was done in HNSCC (HN8), very few cells stained with Annexin V, a close up of the cells that stain for Annexin V showed no colocalization with TCC

To investigate the role of the complement system in HNSCC, we first tested complement activation reflected by deposition of complement components C3 and TCC in 4 patient-derived HNSCC cell lines, and compared the activation to primary human epidermal keratinocytes from adult donors. Using immunofluorescence microscopy, we found that incubation with NHS as a source of complement but not HIS lacking complement activity, led to a significant increase in TCC deposition in HNSCC cells in comparison to primary keratinocytes (Fig. 1a). Interestingly, pooled quantification of C3 and TCC deposition, obtained by subtracting the fluorescence signal of cells treated with NHS from those treated with HIS, showed no significant difference between HNSCC and primary keratinocytes in C3 deposition, but only in TCC staining (Fig. 1b).

The significant increase in TCC staining in HNSCC cells prompted us to investigate the expression of complement regulatory proteins (CRP) CD46, CD55, CD59 and Factor H which are important in regulating complement activation at the cell surface. Using R-T PCR, no significant difference was seen in CD46 and CD55 expression, but CD59 expression - mainly responsible for inhibition of TCC formation [39]- was significantly lower in HNSCC when compared to primary keratinocytes, A431 and A549 (Fig. 1c). Factor H was expressed more in HNSCC cell lines in comparison to primary keratinocytes which had barely detectable levels of Factor H (Fig. 1c). This suggested that the decrease in CD59 expression could be the reason for the observed increased deposition of TCC in HNSCC.

While complement activation was heterogeneously found across the monolayer (Fig. 1a), we noticed that cells with dysmorphic nuclei did not show a similar pattern of complement activation (sup. Fig. 1). So we investigated apoptosis and necrosis in the cell monolayers using Annexin V which binds to phosphatidylserine, a marker of apoptosis when it is on the outer leaflet of the plasma membrane, and Ethidium homodimer III (EtD-III) which is a highly positively charged nucleic acid probe, impermeant to live or apoptotic cells, but stains necrotic cells. We found very few positive cells for apoptosis or necrosis (less than 5%) (Fig. 1d), this is comparable to what we noticed before in primary keratinocytes incubated with NHS [40], such cells had condensed nuclei as shown by DAPI staining (Fig. 1d, arrow) and are similar in nuclear morphology to cells with a different complement deposition pattern (Sup Fig. 1).

To further validate that the observed complement activation was not related to apoptosis, we performed a double stain with Annexin V and TCC, and while most cells were negative for Annexin V staining, we found faint annexin V staining in cells with normal nuclear morphology (Fig. 1e) in HNSCC cell line (HN8), but cells stained with Annexin V showed less TCC staining, and the staining did not colocalize with TCC as C3 often does (Fig. 1a), further emphasizing that complement activation is not associated with phosphatidylserine or apoptotic cells.

In aggregate these data indicate that viable HNSCC cells activate the complement system to a greater extent than healthy epithelial cells as seen by the increase in TCC staining that was paralleled by a decrease in CD59 expression.

### EGFR inhibition and complement activation

To examine if EGFR is involved in regulation of complement activation, we first confirmed the expression of EGFR using R-T PCR and found no significant differences in normalized EGFR expression between the 4 HNSCC cell lines (sup Fig. 2a). As we wanted to study the role of EGFR in regulation of complement activation, we inhibited EGFR using the tyrosine kinase inhibitor Iressa, an FDA approved EGFR inhibitor, rather than using the monoclonal anti-EGFR antibody Cetuximab, which is reported to activate complement through antigen-antibody complexes [41]. The HNSCC response to Iressa was first tested at different concentrations and time points (sup Figure 2b). The average growth inhibition following 10 μM Iressa treatment for two of the cell lines HN4 and HN7 was 20 and 24% respectively, while cell lines HN5 and HN8 averaged growth inhibition was 39 and 46% respectively. This indicated that cell lines HN5 and HN8 were more sensitive to EGFR inhibition treatment, in accordance with a previous account that tested the sensitivity of those cell lines to Cetuximab [42]. Cell counts and viability following Iressa treatment were measured concurrently using an automated cell counter and trypan blue staining, it is important to note here that growth inhibition did not affect viability, which averaged above 90% in those cell lines at 10 μM Iressa concentration (the highest concentration used).

**Fig. 2:**
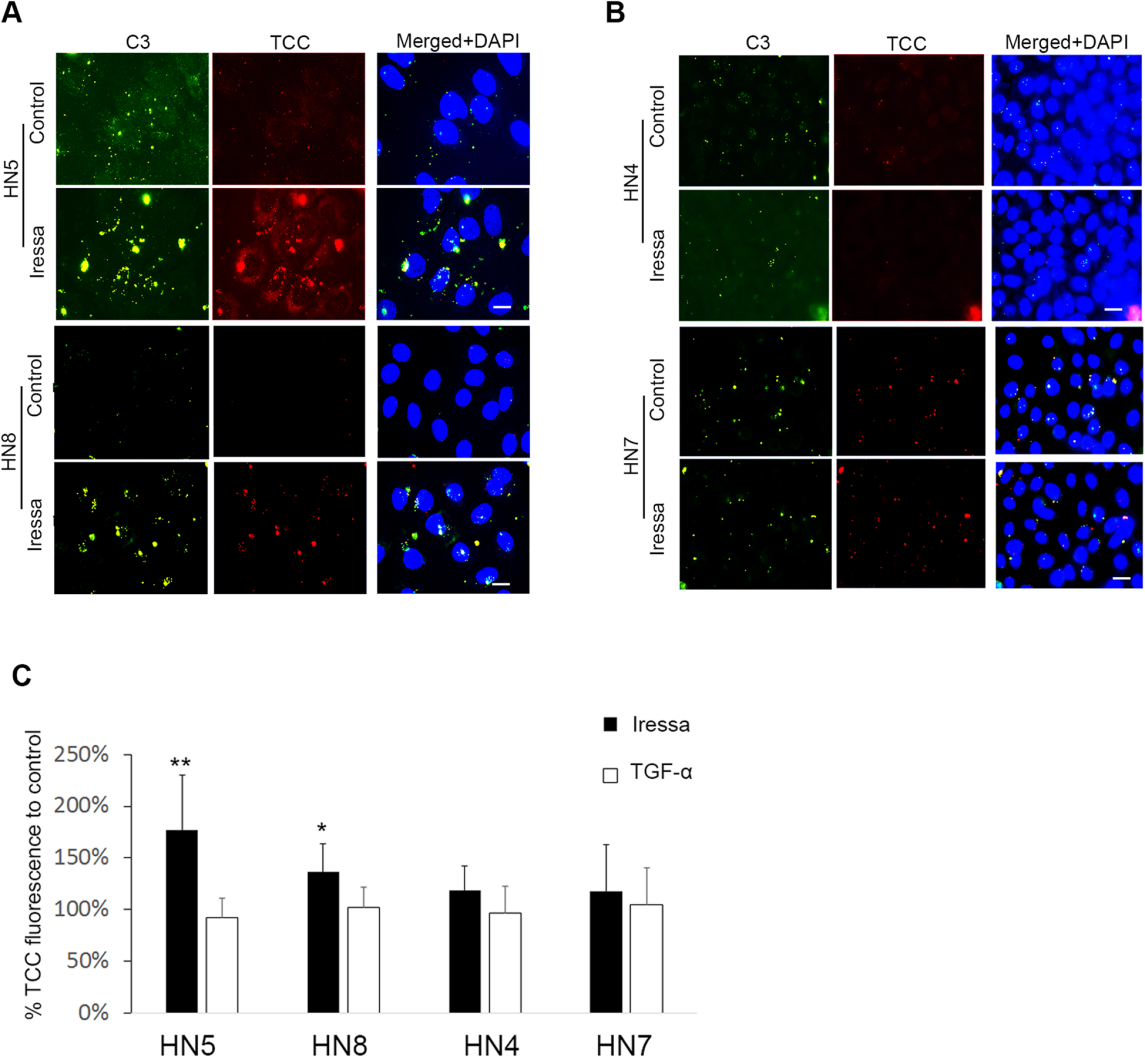
EGFR inhibition leads to complement activation in sensitive cell lines. (**a**) Immunofluorescence microscopy (IFM) shows deposition of complement activation fragments C3 (green) and terminal complement complex TCC (red) on surface of sensitive cell lines HN5 and HN8 after 48 h of Iressa treatment and subsequent incubation with normal human serum (NHS). **(b)** whereas no change in deposition of C3 or TCC is seen in resistant cell lines HN4 and HN7. Heat-inactivated serum (HIS) was used as a negative control. Scale bars, 10 μM. **(c)** IFM quantification of terminal complement complex (TCC) deposition on cell lines after 48 h of EGFR stimulation (TGF-a) or inhibtion (Iressa), and subsequent incubation with NHS. Control incubated with NHS was set to 100%. Error bars represent standard deviation. * *p* < 0.05, ** *p* < 0.01

After confirming the response to EGFR inhibition, HNSCC cells were treated with Iressa for 48 h. In general, we found an increase in TCC deposition in cell lines more sensitive to Iressa HN5 and HN8, the increased deposition of TCC was found after incubation with NHS but not HIS (Fig. 2a,c). In cell lines HN4 and HN7 displaying less growth inhibition, Iressa treatment did not increase TCC deposition after incubation with NHS (Fig. 2b,c). We also investigated if increased EGFR signalling using the potent EGFR ligand TGF-α could affect complement activation but found no significant difference in TCC deposition following transforming growth factor alpha (TGF-α) treatment (Fig. 2c).

**Fig. 3:**
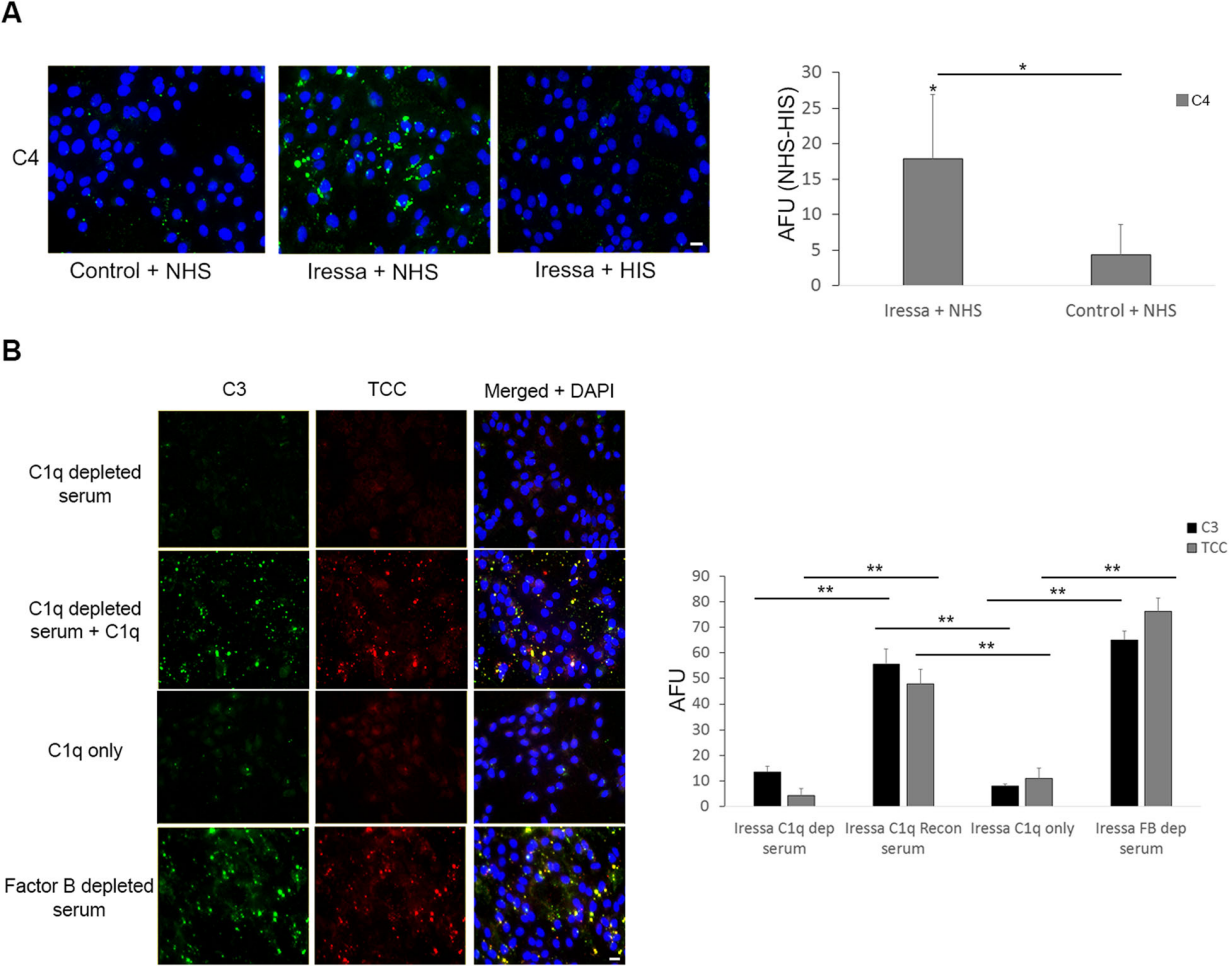
Complement activation after EGFR inhibition is C1q dependent. (**a**) Immunofluorescence microscopy of HN5 cells shows deposition of C4 (green) on surface of Iressa treated cells after incubation with NHS, but not HIS. Controls are not treated with Iressa but incubated with NHS, C4 fluorescence was quantified in the bar chart to the right (**b**) Immunofluorescence microscopy of HN5 cells show no deposition of complement activation fragments C3 (green) and terminal complement complex TCC (red) on surface of HN5 cells after 48 h of Iressa treatment and subsequent incubation in C1q depleted serum, or C1q only (100 μg/mL), but when C1 depleted serum was reconstituted with C1q (100 μg/mL), complement activation was restored. While incubation with Factor B depleted serum, lacking ability to form alternative pathway C3 convertase, still shows complement activation, C3 and TCC fluorescence was quantified in the bar chart to the right. Scale bars, 10 μM. Experiment was repeated three times

To further examine the complement activation mediated by EGFR inhibition on cancer cells, we focused on cell line HN5 with prominent complement activation after EGFR inhibition. Incubation of NHS with Iressa-treated cancer cells led to increased deposition of C4 (Fig. 3a). This indicated activation of the complement system by the classical pathway or lectin pathway. Incubation of Iressa-treated cancer cells with C1q depleted serum, or C1q only, did not lead to complement activation. However, complement activation was found after reconstitution of the C1q-depleted serum with C1q (Fig. 3b). In contrast, incubation with Factor B depleted serum, which lacks an important component in the alternative pathway, led to complement deposition on Iressa-treated HN5 cells (Fig. 3b). This demonstrated that EGFR-inhibition by Iressa treatment led to C1q-dependent complement activation.

Incubation of HN5 cells with radioactive labeled C1q, showed no increased binding of C1q in cells subjected to EGFR inhibition compared to controls, demonstrating that the observed complement activation was not due to increased C1q binding (Sup. Figure 3).

### Complement activation in sensitive cell lines made resistant to EGFR inhibition following prolonged cetuximab treatment

**Fig. 4:**
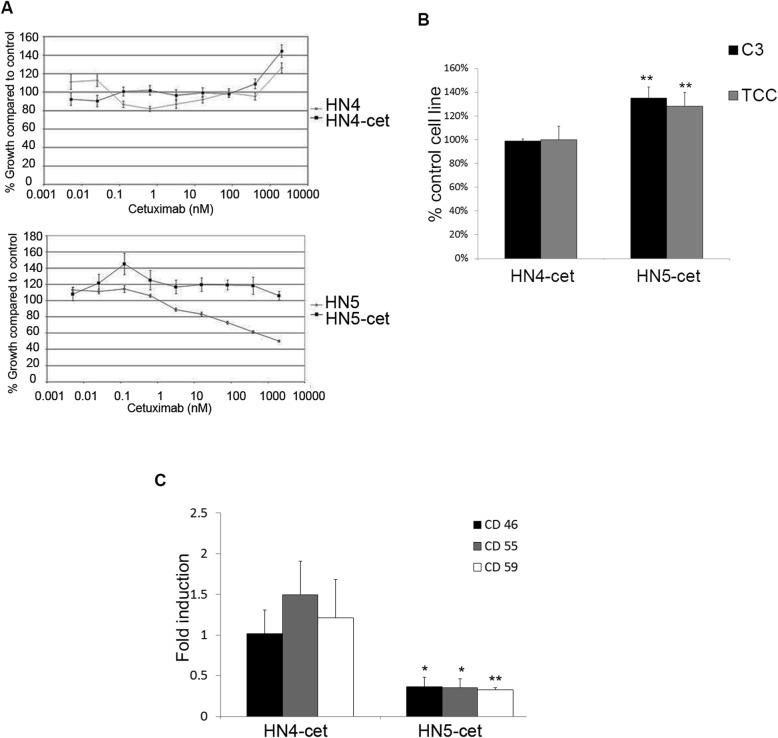
Complement activation and expression of CRP in cetuximab resistant sublines. (**a**) HNSCC cell lines HN4 and HN5 were treated with increasing concentrations of cetuximab to yield resistant sublines HN4-cet and HN5-cet. (**b**) Using IFM deposition of C3 and TCC after incubation with NHS was assessed in HN4-cet and HN5-cet, and compared to their respective original cell lines set to 1. Bars represent the average of 10 representative fields. Error bars represent SEM. (**c**) Expression of CRP in resistant sublines HN4-cet and HN5-cet, was compared to the expression in the original cell lines HN4 and HN5 set to 1, using qPCR. Bars represent the average of 3 experiments. Error bars represent SEM. * *p* < 0.05, ** *p* < 0.01

The therapeutic effect of EGFR inhibition in cancers like HNSCC is mostly short-lived. Consequently, the cell lines were made resistant to EGFR inhibition with prolonged treatment with the EGFR neutralizing antibody cetuximab. The cells subjected to prolonged cetuximab treatment were passaged at least three times in medium without cetuximab to avoid cetuximab induced complement activation. After prolonged cetuximab treatment, the cell line HN5 previously sensitive to EGFR inhibition, did no longer display growth retardation following EGFR inhibition by neither cetuximab (Fig. 4a), nor Iressa (sup Fig. 2b). Interestingly, the HN5 cells made resistant to EGFR inhibition by prolonged cetuximab treatment (termed HN5-cet), now displayed a higher ability to activate complement than the original cell line (Fig. 4b). Moreover, expression of CRP CD46, CD55 and CD59 was significantly reduced in the resistant subline HN5-cet, compared to the original cell line HN5 (Fig. 4c). This demonstrated that prolonged EGFR-inhibition could promote complement activation independent of growth inhibition.

The cell line HN4, previously resistant to EGFR-inhibition, was subjected to similar prolonged cetuximab treatment. In HN4-cet, no increase in the ability to activate complement was found, and the prolonged treatment with cetuximab did not decrease the expression of CRP (Fig. 4a-c).

### Complement activation and ERK phosphorylation

**Fig. 5:**
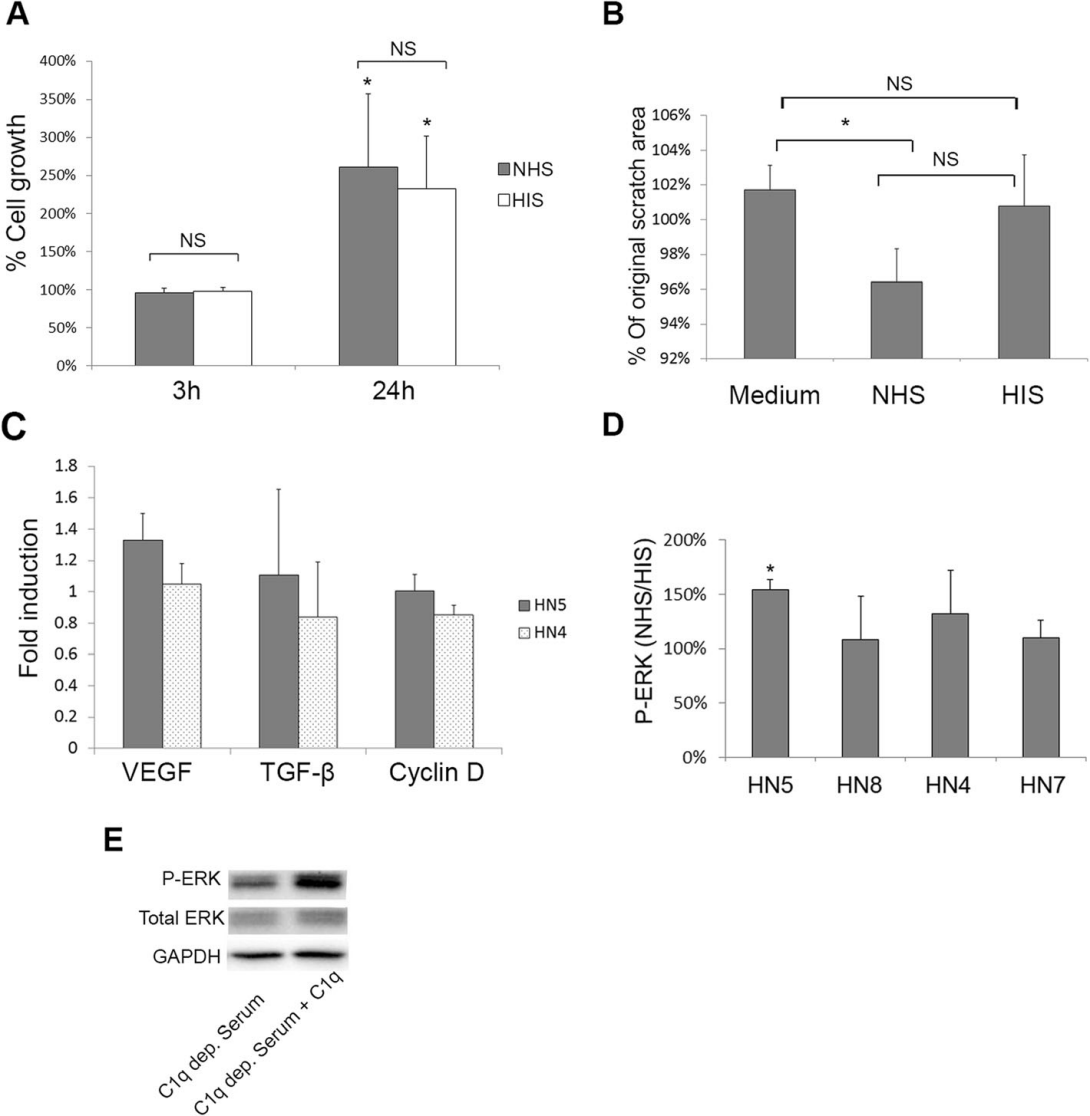
Growth, migration and ERK-activation after complement activation. (**a**) Growth of sub-confluent HN5 cells was measured at 3 and 24 h after adding NHS or HIS, using an SRB assay. Growth of control cells treated with medium only is set to 100%. No significant difference is found between HIS and NHS treatment, indicating complement activation did not affect growth. (**b**) A scratch was made in a monolayer of HN5 cells, and migration of cells into the open area was measured 24 h after adding NHS, HIS or medium only. The area of the original scratch at 0 h is set to 100%. (**c**) Using qPCR, induction of growth and immune related genes was tested after 48 h of Iressa treatment and a subsequent 3 h complement activation using NHS. In cell lines HN5 and HN4, no significant difference in induction is seen when compared to HIS set to 1. (**d**) HNSCC cell lines were treated with Iressa for 48 h and subsequently incubated with 10% NHS or HIS, cell lysates were then blotted for phosphorylated ERK (P-ERK) and normalized to total ERK (T-ERK). Bars represent the ratio of ERK phosphorylation after incubation with NHS compared to HIS, average of 3 experiments is used. (**e**) ERK phosphorylation was further tested in HN5 cells after incubation with C1q depleted serum, and C1q depleted serum reconstituted with C1q (100 μg/mL),and was shown with a representative blot. Error bars represent SEM, * *p* < 0.05

Complement activation fragments have been shown to drive several pro survival signals such as ERK phosphorylation [26], important in cell proliferation and migration [43]. Using cell lysates after complement activation, we found an increase in phosphorylation of ERK1/2 after incubation of cells with NHS compared to HIS in cell line HN5, which had the most prominent complement activation after EGFR inhibition by Iressa, but not in other cell lines (Fig. 5d). To further illustrate the complement role in ERK phosphorylation, we incubated the HN5 cells with C1q depleted serum, or C1q depleted serum reconstituted with C1q. Addition of C1q to C1q depleted serum increased ERK1/2 phosphorylation demonstrating that Iressa-mediated complement activation increased ERK activation in the HN5 cell line (Fig. 5e). This indicates that complement activation observed can be beneficial for the cancer since it promoted ERK phosphorylation, which in addition to its role in cell proliferation, can promote resistance to complement lysis [44].

### Complement activation did not affect growth or migration

Furthermore, we investigated the effect of complement activation on cell growth, cell migration, and growth factor expression in HN5 cells. Using the SRB assay cellular growth was measured 3 and 24 h after addition NHS or HIS to cells at 20% confluency. These cells displayed complement activation after incubation with NHS (data not shown). No significant difference in growth between cells treated with NHS or HIS was observed, indicating complement activation did not affect growth significantly (Fig. 5a).

We also investigated if the observed complement activation affected cell migration, using a scratch assay we measured cell migration in the presence of the growth inhibitor Iressa, to minimize the effect of cell growth on the assay. Though there was a difference between migration of cells after NHS compared to HIS treatment, this was not statistically significant (Fig. 5b). Finally, we examined the expression of growth-related genes possibly induced by complement activation in HN5, using the cell line HN4 as a control. We found no significant difference in gene induction between the two cell lines and hence no significant induction by complement activation (Fig. 5c).

## Discussion

The aberrant growth of epithelial cancers commonly involves EGFR overexpression and activation. Consequently, EGFR inhibition with monoclonal antibodies or tyrosine kinase inhibitors is an FDA approved cancer therapy [45]. Apart from growth, EGFR regulates immune processes like production of chemokines and antimicrobial peptides [46], complement component expression and activation in the epidermis [47], and maintenance of skin homeostasis [48]. Accordingly, many patients undergoing EGFR inhibition therapy develop cutaneous toxicities, which serve as the best prognostic marker for treatment response [45]. The relationship between EGFR signalling and modulation of the immune response prompted us to investigate how EGFR inhibition therapy could affect an important player in the immune response to cancer, the complement system.

We found that viable HNSCC cells activated complement when incubated with NHS. The activation led to increased deposition of TCC when compared to primary keratinocytes. The increased TCC deposition could be explained by our finding of lower CD59 expression in HNSCC when compared to primary keratinocytes. Other reports confirm the presence of elevated systemic complement activation fragments in patients with HNSCC [21], as well as local complement activation [49]. Our finding is in contrast to previous reports indicating elevated CD59 in HNSCCs when compared to non-neoplastic epithelium using immunohistochemistry [50]. This difference could be to the heterogeneity of HNSCC types [51] or the fact that we looked at the relative mRNA levels and not at the protein level.

The only prior studies looking at EGFR and complement activation were investigating the complement fixing potential of monoclonal antibodies like Cetuximab, and the consequent complement dependent cytotoxicity [23, 52]. In this study, we performed complement activation assays with the tyrosine kinase inhibitor Iressa, using HNSCC cell lines with varying growth sensitivities to EGFR inhibition with Iressa. To the best of our knowledge, this has not been previously investigated. We found deposition of complement components C3, C4 and TCC in these cell lines in a manner correlating with the growth inhibitory effect induced by EGFR inhibition. Indeed, cell lines that were more sensitive to EGFR inhibition showed a higher degree of complement activation following Iressa treatment than the more resistant cell lines. This activation was C1q dependent, since C1q-depleted serum did not lead to deposition of complement components, but reconstitution of the depleted serum with C1q did. This could give an extra level of control over complement activation since C1q can be synthesized locally by immune cells found in HNSCC.

Since resistance following prolonged EGFR inhibition treatment commonly takes place in HNSCC, we generated cell lines that were resistant to EGFR inhibition treatment through continuous culture with increasing cetuximab concentrations. We found that the resistant subline significantly decreased CRP expression when compared to the original sensitive cell line. Moreover, incubation of the resistant subline with serum, demonstrated increased C3 and TCC deposition in comparison to the original sensitive cell line, indicating complement activation in the absence of growth retardation by an EGFR inhibitor.

Since inflammation in the tumour microenvironment is commonly modulated to benefit the tumour [31], and the observed complement activation was not due to apoptosis of EGFR inhibited cells, we hypothesize that cancer cells may modulate complement activation during growth inhibitory conditions. Although consequences of complement activation are complex and include interactions with several cell types and immune mechanisms [7], we found direct effects of this complement activation on the state of ERK1/2 phosphorylation, which drives mitogenic events in malignant cells and is reported to play a role in resistance to complement induced lysis [43]. However, we found no statistically significant effect of complement activation on cell growth, cell migration and growth factor expression by comparing cell incubated with NHS and HIS. This may be due to the presence of multiple growth factors in serum.

Inflammation is the best prognostic marker that a tumour is responding to EGFR inhibition therapy. However, the effects of EGFR inhibition on tumours is often short lived [45]. In this context, it is interesting to note that tumour cells that responded to EGFR-inhibition by growth retardation, promoted complement activation that led to ERK-activation. Furthermore, when sensitive cell lines became resistant to EGFR-inhibition, complement activation was present at the cell surface even after termination of EGFR inhibition. It is thus tempting to hypothesize that EGFR-mediated complement activation may, in certain situations, may promote tumour growth.

Activation and regulation of the complement system need to be further investigated in cancers, especially in light of recent evidence that complement inhibitors can be used alongside cancer therapy to improve outcome [53, 54]. Future in vivo experiments using tumour xenografts with varying sensitivities to EGFR inhibition, will hopefully elucidate the local complement response to EGFR inhibitors in the tumour microenvironment.

## Conclusions

Our data demonstrate a complement modulatory role for EGFR in HNSCC. HNSCC cell lines activated complement when incubated with NHS, this activation was increased following EGFR inhibition in cell lines sensitive to Iressa. Complement activation was C1q-dependent, was accompanied by a decrease in CRP, and led to increase ERK phosphorylation in one cell line. In cell lines resistant to Iressa, no increase in complement activation or decrease in CRP was found following EGFR inhibition. Prolonged EGFR-inhibition treatment in cancer cells sensitive to EGFR inhibition led to increased complement activation. This has implications in understanding the response to EGFR inhibitors in cancer treatment where resistance and inflammatory skin lesions are two major causes for treatment cessation.

## Supplementary information

**Additional file 1.** Supplementary Fig. 1. Representative images of cells with dysmorphic nuclie in monolayers. (a) Immunofluorescence microscopy (IFM) showed deposition of complement on cells with normal nuclei, while a cell with a dysmorphic nucleus in the same field showed decreased complement deposition. (b) Differential interference contrast (DIC) image shows a shrunken cell with a different pattern of complement staining as shown with IFM than cells with normal morphology. (c) Superimposed DIC and IFM images shows that cells with abnormal morphology and nuclei do not deposit complement as cells with normal morphology. Supplementary Fig. 2. EGFR expression and sensitivity to Iressa. (a) qPCR measured normalized EGFR mRNA in 4 HNSCC cell lines, each triangle represents a monolayer. (b) Growth inhibition following 5 μM and 10 μM Iressa treatment was measured at 24 h and 48 h, and the average is represented for each cell lines in the bar graph. Uninhibited control growth is set to 100%. Supplementary Fig. 3. Radioactive C1q binding assay was performed on HN4 and HN5 cell lines, after 48 h of EGFR inhibition using 10 μmol/L Iressa. No significant difference in binding between control and Iressa treated cells was found

## Data Availability

All data generated or analysed during this study are included in this published article and its supplementary information files.

## Abbreviations

BSA: Bovine serum albumin
CRP: Complement regulatory proteins
EGFR: Epidermal growth factor receptor
ERK: Extracellular signal-regulated kinase
EtD-III: Ethidium homodimer III
FBS: Foetal bovine serum
HIS: Heat-inactivated human serum
HNSCC: Head and neck squamous cell carcinoma
HRP: Horse radish peroxidase
NHS: Normal human serum
PVDF: Polyvinylidene difluoride
R-T PCR: Real-time polymerase chain reaction
TCA: Trichloroacetic acid
TCC: Terminal complement complex
