# Supplementary material for: EGFR modulates complement activation in head and neck squamous cell carcinoma
Source: BMC Cancer. 2020 Feb 13;20:121. doi: 10.1186/s12885-020-6615-z (PMC7020369; doi:10.1186/s12885-020-6615-z)
Supplement: Supplementary file 1 — Additional file 1. Supplementary Fig. 1. Representative images of cells with dysmorphic nuclie in monolayers. (a) Immunofluorescence microscopy (IFM) showed deposition of complement on cells with normal nuclei, while a cell with a dysmorphic nucleus in the same field showed decreased complement deposition. (b) Differential interference contrast (DIC) image shows a shrunken cell with a different pattern of complement staining as shown with IFM than cells with normal morphology. (c) Superimposed DIC and IFM images shows that cells with abnormal morphology and nuclei do not deposit complement as cells with normal morphology. Supplementary Fig. 2. EGFR expression and sensitivity to Iressa. (a) qPCR measured normalized EGFR mRNA in 4 HNSCC cell lines, each triangle represents a monolayer. (b) Growth inhibition following 5 μM and 10 μM Iressa treatment was measured at 24 h and 48 h, and the average is represented for each cell lines in the bar graph. Uninhibited control growth is set to 100%. Supplementary Fig. 3. Radioactive C1q binding assay was performed on HN4 and HN5 cell lines, after 48 h of EGFR inhibition using 10 μmol/L Iressa. No significant difference in binding between control and Iressa treated cells was found [file 12885_2020_6615_MOESM1_ESM.docx]

## Supplementary material


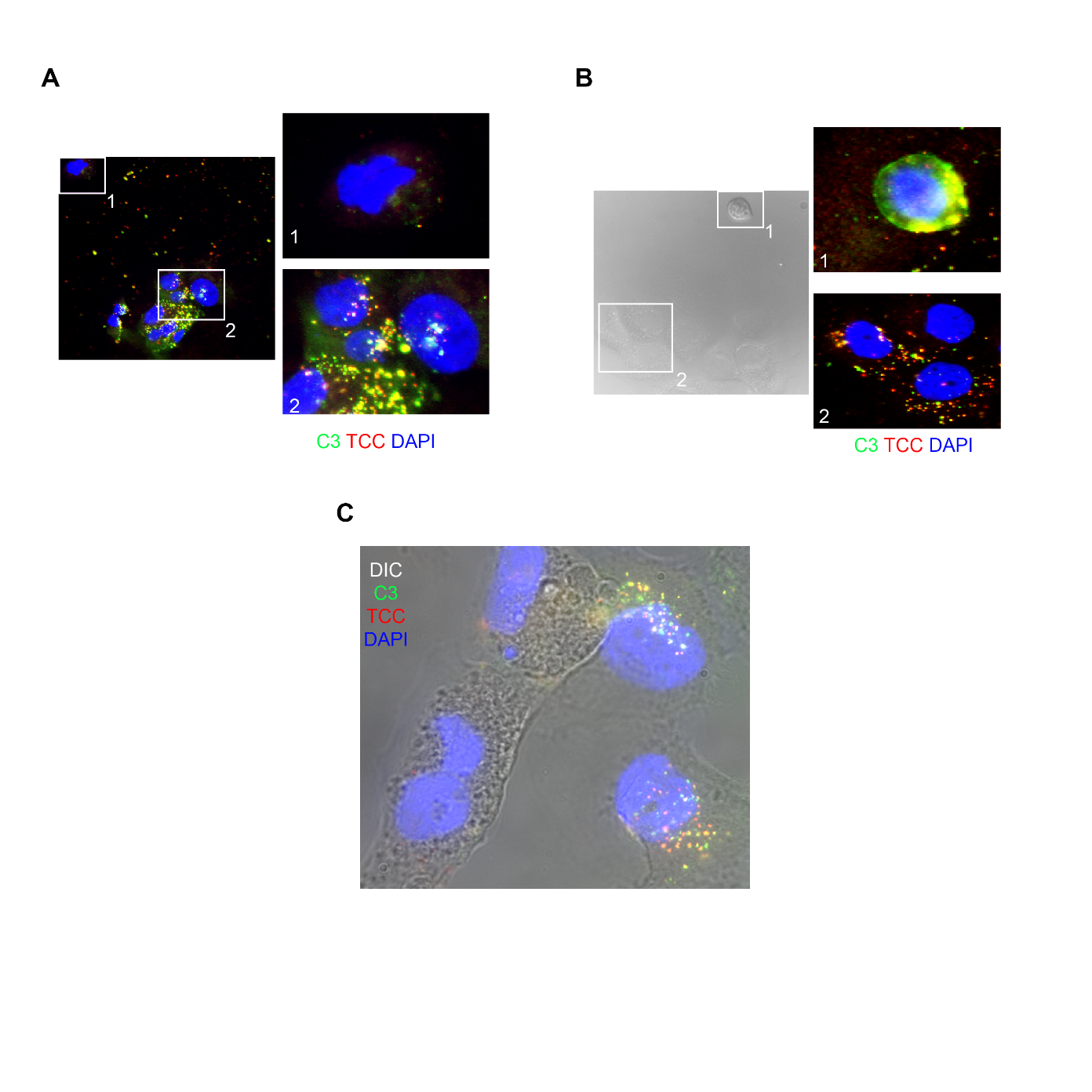


**Supplementary figure 1. Representative images of cells with dysmorphic nuclie in monolayers. (a)** Immunofluorescence microscopy (IFM) showed deposition of complement on cells with normal nuclei, while a cell with a dysmorphic nucleus in the same field showed decreased complement deposition. **(b)** Differential interference contrast (DIC) image shows a shrunken cell with a different pattern of complement staining as shown with IFM than cells with normal morphology. **(c)** Superimposed DIC and IFM images shows that cells with abnormal morphology and nuclei do not deposit complement as cells with normal morphology.

**
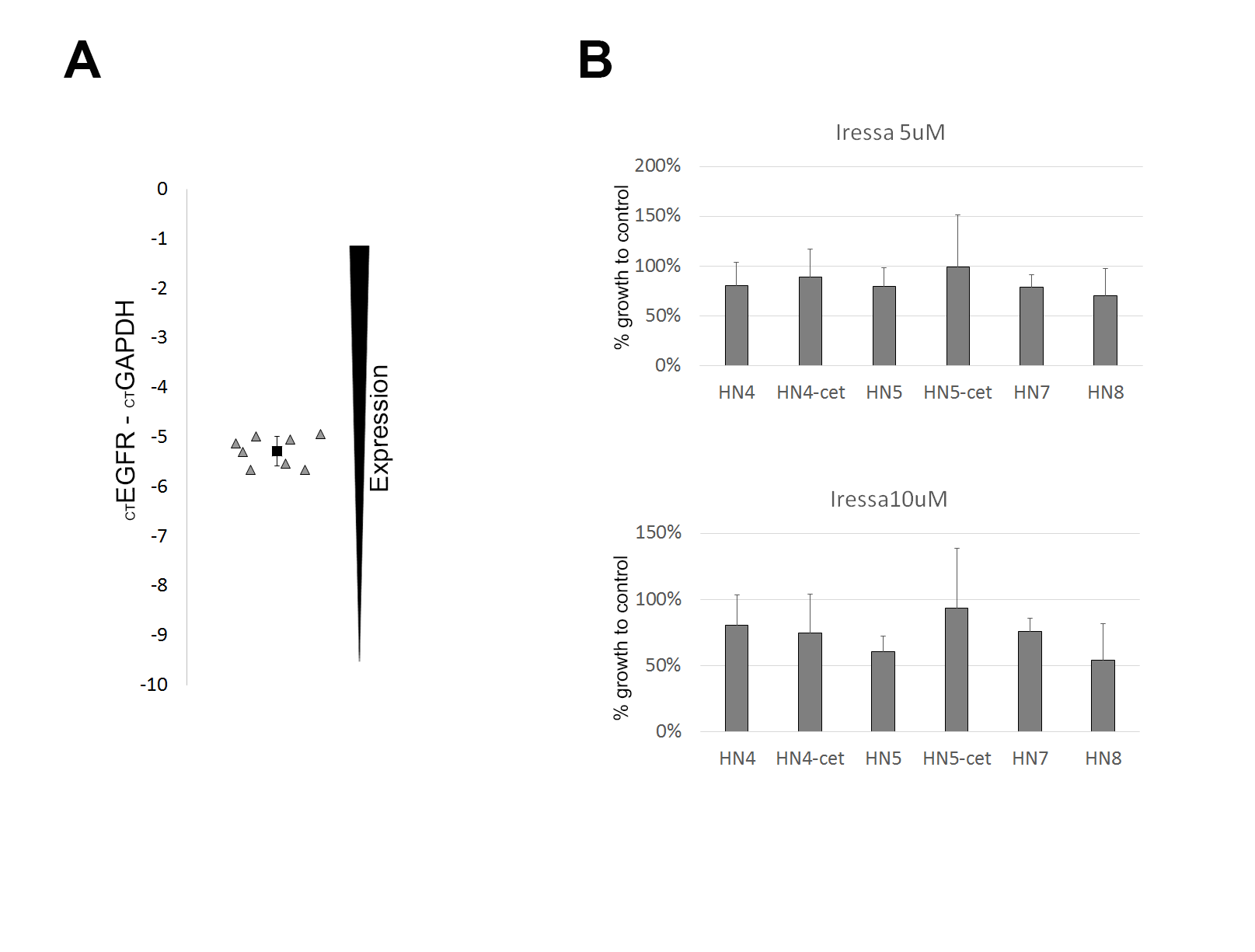
**

**Supplementary figure 2. EGFR expression and sensitivity to Iressa. (a)** qPCR measured normalized EGFR mRNA in 4 HNSCC cell lines, each triangle represents a monolayer. **(b)** Growth inhibition following 5 µM and 10 µM Iressa treatment was measured at 24h and 48 h, and the average is represented for each cell lines in the bar graph. Uninhibited control growth is set to 100%.


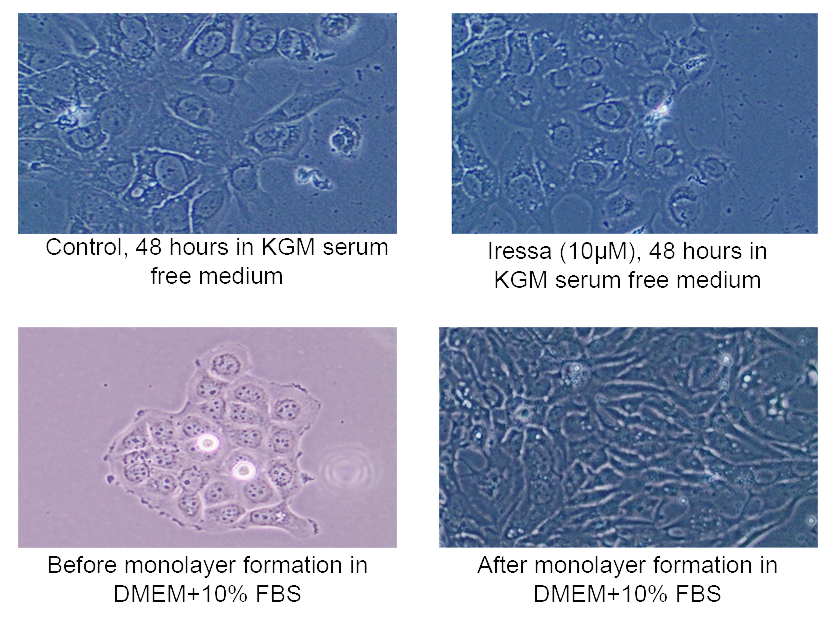


**Supplementary figure 3. Light microscopy of HN5 cells.** Cell morphology was examined using light microscopy at every step following any manipulation of the cell lines. EGFR inhibitors did not induce observable morphological changes. A change in morphology was noted following confluence of the monolayer and changing the DMEM medium to serum free KGM in both EGFR inhibitor-treated and non-treated cells.


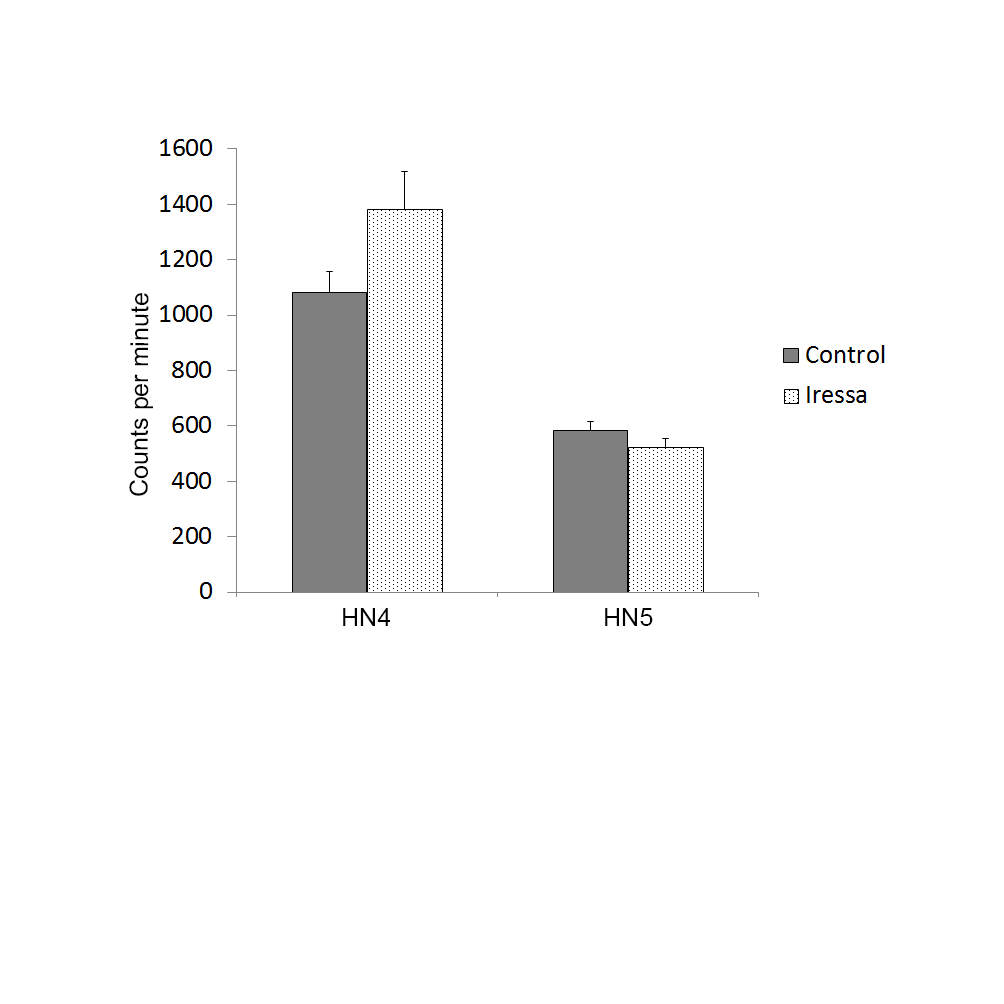


**Supplementary figure 4.** Radioactive C1q binding assay was performed on HN4 and HN5 cell lines, after 48 hours of EGFR inhibition using 10µmol/L Iressa. No significant difference in binding between control and Iressa treated cells was found.
